# T cell–driven venetoclax resistance in chronic lymphocytic leukemia: *in vivo* preclinical activity of opaganib

**DOI:** 10.3389/fimmu.2026.1968413

**Published:** 2026-10-15

**Authors:** Valeria Judith Sarapura Martínez, Paula Soledad Pérez, Anita Ng, Yu-Ju Chen, Kelly Wong, Chiara Cassarino, Rosario Custidiano, Carolina Mahuad, Miguel A. Pavlovsky, Raimundo Fernando Bezares, Martin Bertini, Mercedes Borge, Mónica Vermeulen, Pablo Elías Morande, Mirta Giordano, Matías Ostrowski, Nicholas Chiorazzi, Shih-Shih Chen, Romina Gamberale

**Affiliations:** 1 Instituto de Medicina Experimental (IMEX), Consejo Nacional de Investigaciones Científicas y Técnicas (CONICET)-Academia Nacional de Medicina, Buenos Aires, Argentina; 2 Instituto de Investigaciones Biomédicas en Retrovirus y SIDA (INBIRS), Universidad de Buenos Aires (UBA)-Consejo Nacional de Investigaciones Científicas y Técnicas (CONICET), Buenos Aires, Argentina; 3 Universidad de Buenos Aires, Facultad de Farmacia y Bioquímica, Departamento de Microbiología, Inmunología, Biotecnología y Genética, Cátedra de Virología, Buenos Aires, Argentina; 4 The Feinstein Institutes for Medical Research, Manhasset, NY, United States; 5 Instituto Alexander Fleming (IAF), Buenos Aires, Argentina; 6 Hospital Alemán, Buenos Aires, Argentina; 7 Fundaleu, Buenos Aires, Argentina; 8 Centro Médico Km 41, Moreno, Buenos Aires, Argentina; 9 Hospital General de Agudos Dr. Teodoro Álvarez, Buenos Aires, Argentina; 10 Universidad de Buenos Aires, Facultad de Medicina, Departamento de Microbiología, Parasitología e Inmunología, Cátedra I de Inmunología, Buenos Aires, Argentina

**Keywords:** chronic lymphocytic leukemia (CLL), opaganib, sphingosine kinase 2, T cells, tumor microenvironment, venetoclax resistance

## Abstract

**Introduction:**

Venetoclax has transformed the treatment of chronic lymphocytic leukemia (CLL), yet therapeutic resistance remains a major clinical challenge. In addition to leukemia cell-intrinsic mechanisms, interactions with the immune microenvironment can profoundly reduce venetoclax sensitivity. We previously identified sphingosine kinase 2 (SPHK2) as a therapeutic vulnerability underlying T-cell-supported venetoclax resistance. Here, we investigated the contribution of CD4^+^ and CD8^+^ T cell subsets and their extracellular vesicles (EVs) to this resistant phenotype and evaluated the therapeutic activity of the SPHK2 inhibitor opaganib *in vivo*.

**Methods:**

We evaluated the role of activated CD4^+^ and CD8^+^ T cells and their EVs in the development of the venetoclax-resistant phenotype in primary CLL cells. The *in vivo* activity of opaganib was assessed in a xenograft model generated by co-engraftment of primary CLL cells with autologous activated T cells. Mice were treated with venetoclax, opaganib, or their combination, and leukemic burden and the T cell compartment were evaluated. Anti-apoptotic BCL-2 family proteins were also assessed in residual leukemic cells.

**Results:**

Activated CD4^+^, but not CD8^+^, T cells induced leukemic cell activation and venetoclax resistance, and CD4^+^ T cell-derived EVs were sufficient to reproduce this phenotype. *In vivo*, venetoclax monotherapy failed to reduce leukemic burden, confirming that adaptive interactions with the immune microenvironment are sufficient to sustain therapeutic resistance. Although opaganib alone showed minimal antileukemic activity, its combination with venetoclax reduced splenic leukemic burden by approximately 50% and remodeled the tumor-associated T-cell compartment, without consistent modulation of anti-apoptotic BCL-2 family proteins in residual leukemic cells.

**Discussion:**

These findings are consistent with the notion that the therapeutic effects of opaganib extend beyond leukemic-cell-intrinsic mechanisms and may include modulation of the immune microenvironment. Our study shows that opaganib reduces microenvironment-driven venetoclax resistance in a preclinical CLL model *in vivo*, supporting its clinical evaluation in combination with BCL-2 inhibition.

## Introduction

Chronic lymphocytic leukemia (CLL) is characterized by progressive accumulation of mature clonal B lymphocytes that rely on the anti-apoptotic protein BCL-2 for survival. Venetoclax, a selective BH3 mimetic that binds the hydrophobic groove of BCL-2 and displaces pro-apoptotic proteins, has transformed the therapeutic landscape of CLL. Administered either as monotherapy or in combination with anti-CD20 monoclonal antibodies or Bruton tyrosine kinase inhibitors (BTKi), venetoclax induces deep and durable responses in both clinical trials (1–3) and real-world settings (4). Nevertheless, disease progression eventually occurs in a substantial proportion of patients, and therapeutic options following venetoclax failure remain limited and are frequently associated with poor clinical outcomes (5, 6).

Accumulating evidence indicates that therapeutic response in CLL is determined not only by leukemia cell-intrinsic features but also by the immune context in which leukemic cells reside. Within lymph nodes and other lymphoid tissues, CLL cells are exposed to a highly supportive immune microenvironment composed of activated T lymphocytes, stromal cells, myeloid cells, and other accessory populations that collectively regulate leukemic cell survival, proliferation, and drug responsiveness (7, 8). Rather than serving as a passive niche, this adaptive immune microenvironment actively reshapes apoptotic priming by inducing the expression of alternative anti-apoptotic proteins, including BCL-XL and MCL-1, thereby reducing BCL-2 dependency and limiting the efficacy of venetoclax (9). Although acquired BCL2 mutations such as Gly101Val and Asp103Tyr contribute to clinical resistance, they explain only a subset of relapsing cases (10). Instead, single-cell multiomic studies indicate that resistance evolves through coordinated genetic, transcriptional, and microenvironmental adaptations, emphasizing that venetoclax resistance is a dynamic and multifactorial process rather than the consequence of a single genetic event (10). Importantly, circulating CLL cells recently emigrated from the lymph node supportive microenvironment *in vivo* overexpress multiple anti-apoptotic BCL-2 family proteins even in treatment-naïve CLL patients, and these cells are enriched during venetoclax therapy, highlighting the contribution of the tumor microenvironment to the emergence of venetoclax-resistant leukemic subclones (9, 11).

Among the immune components of the CLL microenvironment, activated T lymphocytes have emerged as particularly potent regulators of leukemic cell fate (12). Beyond promoting leukemia cell activation and proliferation, activated T cells modify apoptotic dependency, thereby influencing sensitivity to BH3 mimetics. We previously demonstrated that autologous activated T cells induce a venetoclax-resistant phenotype through both contact-dependent and contact-independent mechanisms (13, 14). This phenotype is characterized by increased BCL-XL and MCL-1 expression, enhanced leukemic-cell activation and proliferation, and marked resistance to venetoclax-induced apoptosis (14), resembling the microenvironment-driven venetoclax resistant subclones described by Jayappa et al. following leukemic-cell interactions with the lymphoid microenvironment *in vivo* (11).

Extracellular vesicles (EVs) have recently emerged as additional mediators of this bidirectional communication. Stromal-derived EVs decrease CLL cell spontaneous apoptosis and increase their chemoresistance to several drugs, including fludarabine, ibrutinib, idelalisib and venetoclax *in vitro*, while CLL-derived EVs favor the migration, survival and proliferation of stromal cell (15). In addition, CLL-derived EVs show different proteomic profiles during disease progression (16) and their secretion is increased upon BCR signaling (17). Interestingly, CLL-derived EVs also affect the T cell compartment favoring tumor-supportive phenotypes and suppressing antitumor immunity (18, 19). Whether T cell-derived EVs can activate CLL cells and transmit a venetoclax-resistant phenotype remains unknown.

We recently identified SPHK2 as a potential therapeutic vulnerability in T cell-driven venetoclax resistance *in vitro*. CLL cells that acquired venetoclax resistance following interaction with activated T cells consistently exhibited increased SPHK2 expression (20). Pharmacological inhibition of SPHK2 with opaganib not only impaired the development of venetoclax resistant phenotype by reducing T cell-induced BCL-XL upregulation but also re-sensitize already resistant CLL cells to a second venetoclax treatment (20). Together, our *in vitro* findings highlight SPHK2 inhibition as a promising therapeutic strategy to both prevent the emergence of and eliminate established T cell-driven venetoclax resistance.

Opaganib (ABC294640) is an orally bioavailable, SPHK2 inhibitor that has been safely administered to patients with advanced solid and hematological malignancies as well as severe COVID-19 (21–23) and, in preclinical studies, opaganib restores the sensitivity of BRAFV600E-mutant colon cancer cells to vemurafenib *in vitro* (24) and reduces the growth of neuroblastoma cells *in vivo* (25). Moreover, recent studies showed that opaganib induces immunogenic cell death and enhances the efficacy of immune checkpoint blockade in immunocompetent tumor models, supporting an immunomodulatory role beyond its direct antitumor activity (26). While we previously reported the ability of opaganib to overcome T cell-driven venetoclax resistance *ex vivo* (20), it remains unknown whether this immune-mediated resistance phenotype can be faithfully recapitulated in an *in vivo* model of CLL and, if so, whether opaganib can overcome the resulting microenvironment-driven venetoclax resistance.

In the present study, we first extended our characterization of T cell-mediated venetoclax resistance *ex vivo*, dissecting the relative contribution of CD4^+^ versus CD8^+^ T cell subsets and determining whether T cell-derived EVs are sufficient to reproduce this resistant phenotype. We then moved forward to test venetoclax and opaganib *in vivo* in a xenograft model established in NSG mice by co-transferring leukemic B cells with autologous polyclonally activated T cells (27, 28), encompassing both IGHV-mutated and IGHV-unmutated CLL patients to capture prognostically distinct disease subsets (29, 30). Using this model, we found a T cell-supportive venetoclax resistance *in vivo* and assessed the capacity of opaganib, alone and in combination with venetoclax, to reduce leukemic burden and modify the tumor-infiltrating T cell compartment, providing the first *in vivo* evidence for this combination in CLL.

## Methods

### Reagents and antibodies

RPMI 1640, fetal calf serum (FCS), penicillin, trypsin and streptomycin were purchased from GIBCO (Massachusetts, USA). The Ficoll-Hypaque Plus used for cell separation was purchased from Amersham (Buckinhamshire, UK). BSA used for Ab staining buffer was obtained from Weiner Laboratories (Santa Fé, Argentina). Dimethyl sulfoxide (DMSO) was purchased from Sigma-Aldrich (Dallas, TX). Venetoclax (ABT-199) was purchased from MedKoo Biosciences, Inc (Morrisville, NC, USA). Opaganib was provided by Red-Hill Biopharma. PEG 400 and Tween 80 were from Sigma-Aldrich.

Antibodies for *in vitro* experiments: anti-CD19 (PE-Cy5 or FITC, clone HIB19), anti-CD4 (PE, clone OKT4), anti-CD8 (PE-Cy5, clone HIT8a) were from BD Bioscience Pharmingen. Anti-PD-1 (FITC, clone NAT105), anti-CD86 (PE, clone 2331 FUN-1) were obtained from BioLegend. Purified anti-CD3 (clone UCHT1) were obtained from Beckman Coulter (CA, USA). B-CLL human Microbeads isolation kit was obtained from Miltenyi biotec (Germany).

Antibodies for EV-Western Blot: Anti-human CD63 (clone H5C6, BD Pharmingen). Anti-human CD81 (clone JS-81, BD Pharmingen). Anti-human HSP90B1 (clone 9G10, Enzo Life Sciences). Anti-human CD3z (clone 6B10.2, BioLegend). Anti-human CD40L (clone 40804, R&D Systems).

Antibodies for *in vivo* experiments: Anti-mouse CD45 (APC/Cy7, clone 30-F11, Biolegend). Anti-human CD45 (eFluor 450, clone 2D1, eBioscience, Invitrogen), CD19 (clone HIB19, PE/Dazzle™ 594 from BioLegend and PE from BD Bioscience Pharmingen), CD5 (APC, clone UCTH2, Southern Biotech), CD3 (PECy7, Clone UCHT1, BioLegend), CD4 (PerCP-Cy5.5, clone OKT4, eBioscience, Invitrogen), CD8 (FITC, clone HIT8a, BD Bioscience Pharmingen), PD-1 (PE, clone NAT105, BioLegend), Ki67 (PE/Cy7, clone Ki-67, BioLegend), BCL-2 (AF488, clone 100, BioLegend), MCL-1 (AF594, clone D2W9E, Cell Signaling), BCL-XL (AF700, clone 54H6, Cell Signaling).

### CLL patients

Peripheral blood samples were collected from nineteen unrelated, treatment-naïve CLL patients including nine patients with mutated IGHV and eight with unmutated IGHV. Mutational status was not available for two patients. CLL was diagnosed according to standard clinical and laboratory criteria. All samples used in this study were obtained after informed consent in accordance with the Declaration of Helsinki and with Institutional Review Board approval from the Academia Nacional de Medicina (Buenos Aires, Argentina) or by the Northwell Health Institutional Review Board (NY, USA).

### Cell separation procedures

Peripheral blood mononuclear cells (PBMC) were isolated from fresh blood samples by density centrifugation over a Ficoll-Hypaque Plus gradient, washed twice with saline solution, and resuspended in complete medium (RPMI 1640 supplemented with 10% FBS, 100 U/mL penicillin and 100 µg/mL streptomycin). Cells were used immediately or were cryopreserved in FCS 10% DMSO for further experiments.

CLL B cells were purified using the Human CLL B Cell Isolation Kit (MACS MicroBeads, negative selection; Miltenyi Biotec) according to the manufacturer’s instructions. Cell purity and viability were assessed by flow cytometry following CD19 staining, and a purity of ≥98% was confirmed.

CD4^+^ and CD8^+^ T cells were stained with anti-CD4-PE and anti-CD8-Cy5 monoclonal antibodies and subsequently isolated by fluorescence-activated cell sorting (FACS) using a FACSAria FUSION cell sorter (BD Biosciences). The purity of each sorted population was assessed by flow cytometry, and a purity of ≥98% was confirmed.

### CLL cell activation and venetoclax resistance generation with purified CD4^+^ or CD8^+^ T cells

Purified CLL cells were co-cultured with purified CD4^+^ or CD8^+^ T cells, at a ratio of 2,3 CLL/1 T cells (final density 2x10^6^ cells/ml) in complete culture medium with platebound anti-CD3 (aCD3 0,25 μg/ml, 96-well plate) or the isotype control (control cultures) for 72 h. Venetoclax (0.2 μM) or DMSO (control) were added to the cultures for the last 24 h.

### Isolation of CD4^+^ T cell-derived extracellular vesicles

CD4^+^ T cells isolated from healthy donors or patients with CLL, as described above, were stimulated with anti-CD3/CD28/CD2 activation beads (Miltenyi Biotec) for 48 h in EV-depleted culture medium, supplemented with 10 U/mL recombinant IL-2. Following stimulation, cells and cellular debris were sequentially removed by centrifugation at 300 × g for 10 min and 2,000 × g for 10 min. After each centrifugation step, the supernatant was transferred to a fresh tube and the pellet was discarded. EVs were subsequently pelleted by centrifugation at 30,000 × g for 90 min using a Biofuge Stratos centrifuge equipped with a Heraeus rotor (k-factor = 340). This EV purification protocol has been previously standardized in our laboratory (31) and EVs produced by activated CD4^+^T cells were characterized by scanning electron microscopy (**Supplementary Figure S1A**) and immunoblot (**Supplementary Figures S1B, C**). Briefly, centrifugation at 30,000 × g for 90 min was validated by WB to be sufficient to pellet most EVs in the sample, since a following centrifugation of 30,000 × g supernatant at 100,000 × g did not render significant EV-marker signal. The EV pellet was washed with PBS and centrifuged again under the same conditions (**Supplementary Figure S1C**). EV identity was further confirmed by western blot analysis of the EV markers CD63 and CD81, and the T-cell marker CD3, the molecule CD40L, together with HSP90B1, an endoplasmic reticulum-resident protein used as a negative control for EV preparations and detected only in total cell lysates. A representative western blot is shown in **Supplementary Figure S1B**.

#### Preparation of EV-depleted culture medium

EV-depleted culture medium was prepared by ultracentrifugation of RPMI 1640 supplemented with 30% FBS at 100,000 × g overnight using a Beckman 70Ti rotor (k-factor = 220). The resulting supernatant was subsequently diluted with RPMI 1640 to obtain a final concentration of 10% EV-depleted FBS.

#### CLL cell activation and venetoclax resistance generation with EVs

For EV stimulation experiments, purified CLL cells were cultured alone or in the presence of CD4^+^ T cell derived EVs for 72 h. EVs corresponding to those released by 1x10^6^ CD4^+^ T cells were added to 0,2 x 10^6^ CLL cells. Venetoclax (0.2 μM) or DMSO (control) were added to the cultures for the last 24 h.

### Quantitation of viable cells

Cell viability was evaluated by flow cytometric alterations of light-scattering properties (FSC-H), where apoptotic cells could be easily distinguished from viable cells because of their lower forward light scatter, consistent with reduction of cell size and cytoplasmic volume occurring during apoptosis, as we previously reported (13, 32). In these cases, cell survival was quantified as the percentage of cells within the gate of viable cells. These results were confirmed by Annexin V-FITC staining (not shown).

### CLL xenograft model in alymphoid NSG mice

*In vivo* experiments were conducted at The Feinstein Institutes for Medical Research in Manhasset, New York. All experiments were performed in accordance with the guidelines of the Northwell Health Institutional Animal Care and Use Committee (IACUC). Mice were maintained on a 12:12 hour light/dark regime in a BSL-2 laboratory.

This murine model consists of injecting PBMCs from CLL patients, accompanied by activated autologous T lymphocytes, into NOD-scid IL2Rgammanull mice (“NSG™”, Jackson Laboratories) (27). T lymphocytes were isolated from PBMCs of CLL patients using anti-CD3 microbeads (Miltenyi Biotec), following the manufacturer’s recommended protocol. After confirming a purity greater than 90% by flow cytometry, and with the aim of activating and expanding these T lymphocytes, 1 × 10^6^ CD3^+^ cells/ml, were cultured for 7 to 10 days in culture medium (RPMI-1640 supplemented with 10% heat-inactivated FBS and antibiotics) with Dynabeads CD3/CD28 25 µl/ml (Invitrogen) and IL-2 (30 units/ml; R&D), at 37 °C, 95% humidity, and 5% CO_2_. The cultures were maintained at 1 × 10^6^ cells/ml with fresh culture medium containing IL-2.

After adequate expansion and viability of the T cells were confirmed, activation microbeads were removed using a magnet. Subsequently, PBMCs from the same patient (for each experiment) were thawed and assessed viability with trypan blue, confirming a viability greater than 70% in all cases. Next, 20 × 10^6^ viable PBMCs were combined with activated T lymphocytes (in a 1:40 CD3^+^:PBMC cell ratio) and injected them into 3.5–5.5 weeks old male NSG mice.

Each NSG mouse received 20.5 million live cells resuspended in 100 µl of PBS, transferred intravenously (IV) through the retro-orbital plexus. Isoflurane anesthesia was administered for retro-orbital injections, adhering to Institutional Animal Care and Use Committee (IACUC) guidelines and the American Veterinary Medical Association (AVMA) standards. Mice were given 3% isoflurane in oxygen (2L/min) within a sealed Plexiglas induction chamber. Upon immobilized, mice were promptly transferred to a nose cone for maintenance anesthesia at 1.5% isoflurane during the retro-orbital vein injection procedure. A total of 20 million CLL patient PBMC and 0.5 million autologous T cells, suspended in 100ul PBS, were then injected via the retro-orbital vein. We employed primary cells from 6 treatment-naïve CLL patients, including 3 with mutated IGHV and 3 with unmutated IGHV. Initially 103 mice were injected.

After 2 weeks, peripheral blood samples were collected by mandibular vein puncture. PBMCs were isolated as described above and immunostained with antibodies reactive with murine and human CD45, and human CD19, CD3, and CD5. Samples were then analyzed by flow cytometry. Mice with less than 3% hCD45^+^ cells on peripheral blood on day 14 were excluded from the study (N = 7), therefore 96 mice were finally included in the study.

#### *In vivo* treatment

After confirming the presence of human CLL cells (hCD45^+^CD19^+^CD5^+^cells) in the peripheral blood samples of the injected mice, animals were assigned to the respective treatment groups (**Supplementary Table 1**). Given the possibility of active drug excretion through feces, treatment allocation was performed at the cage level, with all animals housed within the same cage receiving the same treatment, thereby minimizing the potential for cross-exposure between treatment groups.

There were 4 treatment groups. In all cases, the twice-daily treatment was administered by oral gavage at a final volume of 150 µl/dose, for 3 weeks, 5 days a week. The treatment groups were:

1- Control: vehicle (PEG400 46% + Tween 80 6.7% + PBS) in the morning and in the afternoon (12 h apart).

2- Venetoclax/opaganib: Venetoclax + opaganib in the morning and opaganib in the afternoon (12 h apart).

3- Venetoclax: Venetoclax in the morning and vehicle in the afternoon (12 h apart).

4- Opaganib: opaganib in the morning and in the afternoon (12 h apart).

Regarding treatment tolerability, no treatment-related changes in body weight were observed among the four treatment groups (**Supplementary Figure S2**), supporting adequate tolerability based on body weight as an indirect indicator. During the oral gavage procedure, one mouse from the vehicle group in the experiment with CLL #5 was lost; this death was attributed to the gavage procedure rather than to treatment-related toxicity. Overall, 95 animals were ultimately included in the analysis.

#### Drug dosage and preparation

Vehicle: PEG400 46% + Tween 80 6.7% + PBS, 1 h at 60 °C. It was prepared once a week and then stored at room temperature, protected from light.

Venetoclax: diluted in vehicle, at a concentration of 12.5 mg/ml. To achieve proper dilution, magnetic stirring was maintained for 1 hour, sonicating for 1 minute every 20 minutes. This dilution was prepared daily. Dosage: 5 mg/kg/day for the first 3 days, 10 mg/kg/day for the next 3 days, 25 mg/kg/day until the end of the protocol (33).

Opaganib: diluted in vehicle, at a concentration of 37.5 mg/ml. To achieve proper dilution, magnetic stirring was maintained at 50 °C for 4 hours, sonicating for 1 minute every 20 minutes during the last hour. We prepared the solution once a week and then stored it at room temperature, protected from light. Dosage: 150 mg/kg/dose every 12 hours for the first week, and 75 mg/kg/dose every 12 hours for the following two weeks, following the suggestions of RedHill Biopharma, the company that provided the drug.

#### Spleen, bone marrow and peripheral blood assessment

At euthanasia, spleens, bone marrow from both femurs and tibias, and peripheral blood were obtained. Euthanasia was performed in all cases in accordance with IACUC guidelines, aligned with the AVMA standards. As the primary chemical method, carbon dioxide (CO_2_) inhalation was administered in the home cage using a controlled gas flow rate. Our CO_2_ euthanasia stations are set to ensure a volume displacement rate of 30% to 70% of the chamber volume per minute for rodents with a target displacement rate of ~40-50% chamber volume per minute.

The procedure was maintained until the cessation of respiration was confirmed. Cervical dislocation was then performed as a secondary physical method to ensure death.

We prepared cell suspensions from spleens by mechanical disaggregation and posterior red blood cells lysis (ACK). Subsequently, immunolabeling for CD45 (human and murine), CD19, CD5, CD3, CD4, CD8, PD-1, Ki-67, BCL-2, BCL-XL, and MCL-1 (human) were performed. Cell suspensions from bone marrow were also obtained by mechanical disaggregation and then PBMCs were obtained by density gradient in Ficoll. PBMCs from peripheral blood were also obtained by Ficoll density gradient. In both cases, cells were then immunolabeled for CD45 (human and murine), CD3, CD19, and CD5 (human). Data acquisition for flow cytometric measurements was performed using BD LSRFortessa flow cytometer (BD Biosciences). Patient-derived CLL B cells were gated on human CD45^+^CD19^+^CD5^+^ cells and patient-derived T cells were defined as human CD45^+^CD3^+^CD19^−^ cells.

#### Cell count

Absolute cell counts in the collected tissues were determined using absolute count microbeads (Invitrogen). Microbeads were maintained at room temperature for 30 minutes and vortexed for 30 seconds before use. Immediately prior to flow cytometry acquisition, 10 µL of microbeads was added to each cell suspension. Absolute cell counts were calculated according to the manufacturer’s instructions using the following formula:

Absolute count (cells/µl) = (Cell count * Microbeads volume/Microbeads count * Cell volume) * Microbeads concentration (microbeads/µl).

### Statistical analysis

Statistical significance was determined using paired T test and RM-one-way Anova followed by Holm-Sidak’s multiple comparisons test for *in vitro* experiments. *In vivo* experiments statistical analyses were performed employing Kruskal-Wallis test followed by Dunn’s multiple comparisons test. In all cases, p<0.05 was considered statistically significant. Data was analyzed using GraphPad Prism software version 8.4.2. Treatment effects were also estimated with a linear mixed-effects model on log-transformed CD19^+^ cell counts, with treatment as a fixed effect and donor of origin as a random intercept, fitted by restricted maximum likelihood and the three comparisons of each treatment arm against vehicle were Holm-adjusted as a single family. Data was analyzed using R/RStudio (version 2026.09.0 + 174).

## Results

### The role of CD4^+^ and CD8^+^ T cells in CLL activation and T cell-driven venetoclax resistance *in vitro*

To further investigate T cell-mediated venetoclax resistance in CLL cells, purified CLL cells (pCLL) were cultured with autologous purified CD4^+^ (pCD4) or CD8^+^ T cells (pCD8) with anti-CD3 or isotype control for 72 h. Venetoclax at a clinically relevant concentration (0.2 µM), or DMSO as vehicle control, was added during the final 24 h of culture (**Figure 1A**). CLL-cell viability and the expression of the activation markers CD86 and PD-1 were then evaluated by flow cytometry within the CD19^+^ population.

**Figure 1:**
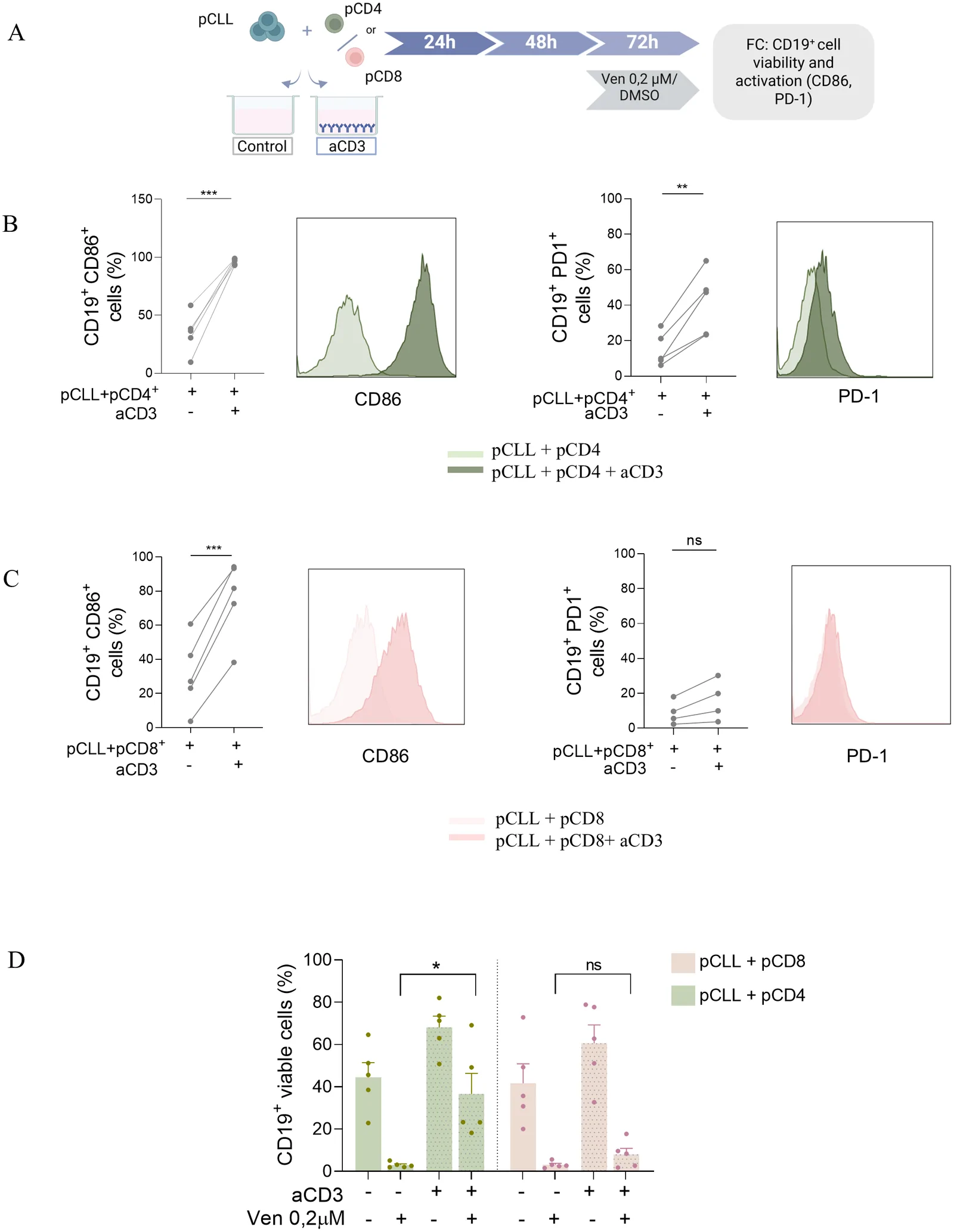
Activated CD4^+^ T cells induce venetoclax resistance on CLL cells. **(A)** Schematic representation of experimental design. Created in BioRender. sarapura martinez, V. (2026) https://BioRender.com/7dsagit. Purified CLL (pCLL) cells were co-cultured with autologous purified CD4^+^ (pCD4^+^) or CD8^+^ (pCD8^+^) T cells in complete culture medium with platebound anti-CD3 (aCD3) or the isotype control for 72 h. Venetoclax (Ven, 0.2 μM) or DMSO (control) were added to the culture for the last 24 h. Activation markers (CD86 and PD-1) on CLL cells and cell viability were assessed by flow cytometry (FC). **(B, C)** The figures show the expression of the activation markers CD86 and PD-1 on CLL cells cocultured with pCD4^+^ **(B)** or pCD8^+^ **(C)** T cells. The graphs show the mean ± SEM of the percentages of CD19^+^CD86^+^ (n=5) and CD19^+^PD-1^+^ cells (n=4). Histograms obtained from the flow cytometry analysis of a representative patient are also shown. Statistical analysis was performed with paired T test, **p<0.01, ***p<0.001, ns p>0.05. **(D)** The graph shows the mean ± SEM of viable CD19^+^ cells in each culture condition. Statistical analysis was performed employing One-way ANOVA with Geisser-Greenhouse correction followed by Holm-Sidak multiple comparisons test * p<0.05, ns p>0.05, n=5.

Activated CD4^+^ T cells induced a clear activation phenotype in CLL cells, as shown by the upregulation of both CD86 and PD-1 (**Figure 1B**). When activated CD8^+^ T cells were evaluated we found that they also promoted CLL-cell activation, as evidenced by increased CD86 expression, but did not significantly alter PD-1 expression on the leukemic clone (**Figure 1C**). Functionally, only activated CD4^+^ T cells conferred resistance to venetoclax-induced cell death, whereas activated CD8^+^ T cells, despite inducing partial activation of CLL cells, did not modify their sensitivity to the drug (**Figure 1D**).

Since we previously demonstrated that secreted factors released by activated T cells can also promote venetoclax resistance in CLL cells (14), we next investigated whether EVs derived from autologous activated pCD4^+^ T cells contributed to this effect. EVs were isolated by differential centrifugation, and their presence was confirmed by scanning and transmission electron microscopy as well as western blot analysis (**Supplementary Figure S1**). Purified CLL cells were then cultured with these EVs for 72 h with venetoclax exposure during the final 24 h of culture (**Figure 2A**). In a limited number of experiments, EVs derived from CD4^+^ T cells from patients with CLL increased the expression of CD86 and PD-1 on autologous CD19^+^ CLL cells (**Figure 2B**) and reduced their sensitivity to venetoclax (**Figure 2C**). Because patient-derived CD4^+^ T cells were limited in availability and because EVs derived from CD4^+^ T cells from healthy donors also reduced CLL cell sensitivity to venetoclax (**Figure 2C**), we subsequently performed additional experiments using EVs from healthy donor pCD4^+^ T cells. As shown in **Figure 2D**, these EVs increased CD86 and PD-1 expression on CD19^+^ CLL cells, suggesting that EV-mediated signals can induce an activated phenotype in the leukemic cells. Moreover, EV exposure reduced CLL cell sensitivity to venetoclax (**Figure 2E**), supporting a role for T-cell-derived EVs in promoting venetoclax resistance in CLL.

**Figure 2:**
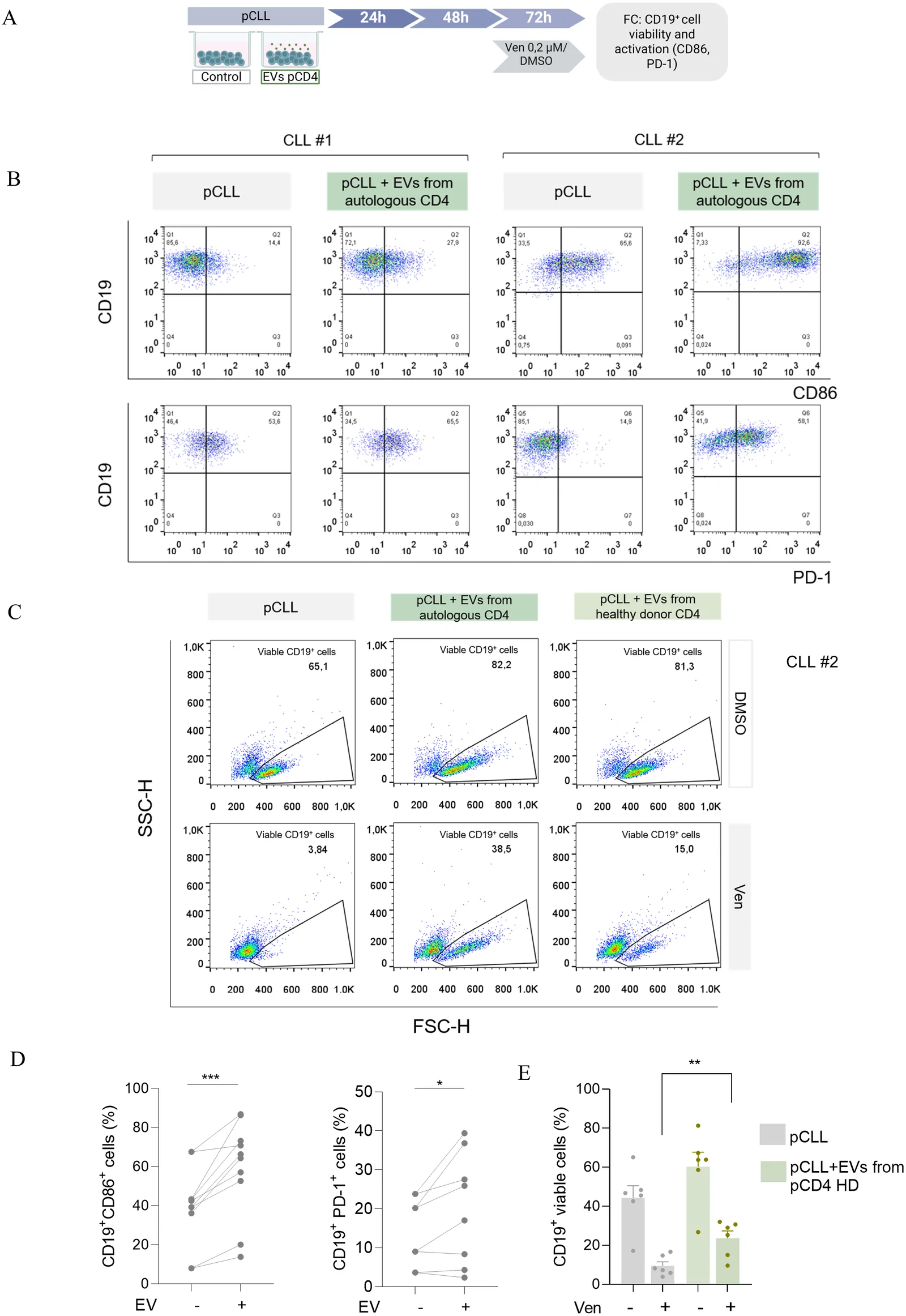
Extracellular vesicles (EVs) derived from activated CD4^+^ T cells promote CLL cell activation and induce venetoclax resistance. **(A)** Schematic representation of experimental design. Created in BioRender. sarapura martinez, V. (2026) https://BioRender.com/zbtg9wb. Purified CLL (pCLL) cells were cultured alone or in the presence of CD4^+^ T cell derived EVs for 72 h. EVs corresponding to those released by 1x10^6^ CD4^+^ T cells were added to 0,2 x 10^6^ CLL cells. Venetoclax (ven, 0.2 μM) or DMSO (control) were added to the cultures for the last 24 h. Activation markers (CD86 and PD-1) on CLL cells and cell viability were assessed by flow cytometry (FC). **(B)** Dot plots obtained by flow cytometry assessment showing CD86 and PD-1 expression on pCLL cells cultured with or without EVs from autologous CD4^+^ T cells from two CLL patients. **(C)** pCLL from one patient were cultured alone or in the presence of EVs derived from autologous CD4^+^ T cells or EVs derived from healthy donor CD4^+^ T cells for 72 h, with venetoclax or DMSO during the last 24 h of culture. Dot plots obtained by flow cytometry (FSC-H, Forward Scatter versus SSC-H, Side Scatter) showing the percentage of cells within the gate of viable cells in each culture condition are shown. **(D, E)** pCLL were cultured alone or in the presence of EVs derived from healthy donors (HD) CD4^+^ T cells as mentioned above. **(D)** The graphs show the mean ± SEM of the percentages of CD19^+^CD86^+^ (n=10) and CD19^+^PD-1^+^ (n=8) CLL cells cultured in the presence or absence of EVs. Statistical analysis was performed with paired T test * p< 0.05, *** p<0.001. **(E)** The graph shows the mean ± SEM of viable CD19^+^ cells in each culture condition. Statistical analysis was performed employing One-way ANOVA with Geisser-Greenhouse correction followed by Holm-Sidak multiple comparisons test, ** p<0.01, n=6.

### Venetoclax resistance induced in a CLL xenograft model can be bypassed leading to reduced tumor burden by the combination of venetoclax with opaganib

To determine whether T cell-mediated venetoclax resistance could be reproduced *in vivo*, we employed a CLL xenograft model in which CLL cells are co-transferred with autologous polyclonally activated T cells into alymphoid NSG mice (27, 28). Using this approach, we further evaluated the ability of the SPHK2 inhibitor, opaganib, to overcome venetoclax resistance *in vivo* as we previously reported *in vitro* (20).

A total of 95 NSG mice were engrafted with PBMC obtained from 6 patients with CLL, including 3 patients with mutated IGHV, representing the favorable prognostic subgroup, and 3 patients with unmutated IGHV, corresponding to the poor-prognosis subgroup (29, 30), together with autologous activated T cells. Two weeks after cell transfer, successful engraftment was confirmed by the detection of human CD45^+^CD19^+^CD5^+^ cells in peripheral blood, and mice were subsequently treated for 3 weeks with the vehicle (control), venetoclax, opaganib, or the combination of venetoclax and opaganib (see Materials and Methods). At the end of the treatment period, mice were euthanized and spleen, bone marrow, and peripheral blood samples were analyzed by flow cytometry (**Figure 3A**).

**Figure 3:**
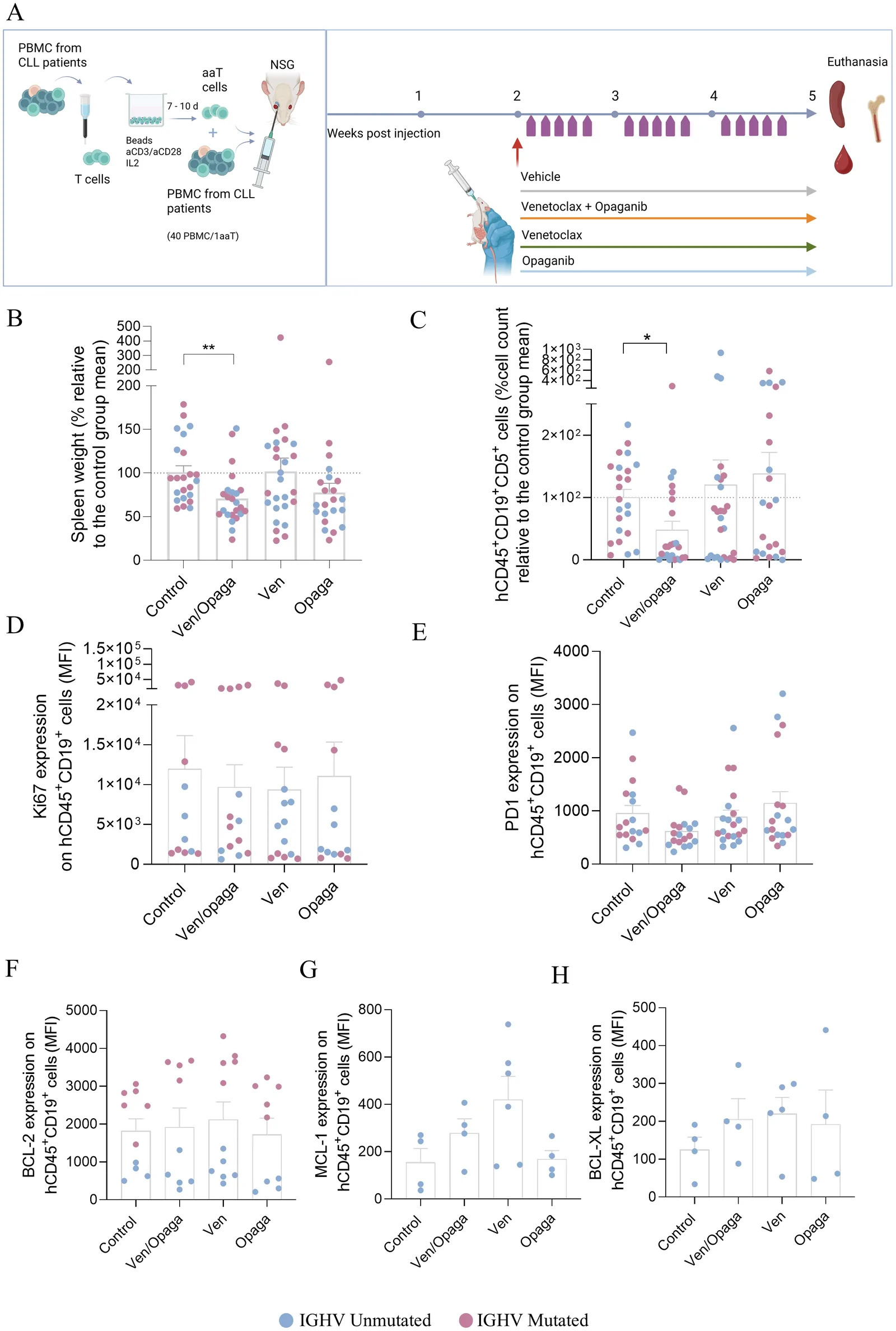
Venetoclax resistance induced in a CLL xenograft model can be reduced by the combination of venetoclax with opaganib. **(A)** Schematic representation of the *in vivo* experimental protocol. Created in BioRender. sarapura martinez, V. (2026) https://BioRender.com/x0vz657. 95 NSG mice were intravenously injected with peripheral blood mononuclear cells (PBMCs) + previously expanded and activated autologous T cells (aaT) in a 40:1 proportion. 6 CLL patients (3 with mutated and 3 unmutated IGHV) were evaluated in 6 independent experiments. Two weeks after cell injection, engraftment was confirmed by detection of human CD45^+^CD19^+^CD5^+^ cells in peripheral blood and mice were randomized to 4 treatment groups: Control (Vehicle: PEG400 46% + Tween 80 6,7% + PBS), Ven/opaga (venetoclax + opaganib), Ven (venetoclax) or Opaga (opaganib). Treatments were administered by oral gavage 5 days a week, for 3 weeks. Subsequently mice were euthanized and spleen, bone marrow, and peripheral blood samples were analyzed by flow cytometry (FC). **(B)** The graph shows the mean ± SEM of the spleens weight relative to the mean of the control group, n=95. **(C)** The graph shows the mean ± SEM of hCD45^+^CD19^+^CD5^+^ cells count in spleens relative to the mean of the control group, n=95. **(D)** The figure shows the mean ± SEM of Ki-67 expression in splenic hCD45^+^CD19^+^ cells from mice across four independent experiments, n=54. **(E)** The figure shows the mean ± SEM of the percentage of splenic hCD45^+^CD19^+^PD-1^+^ cells from mice across four independent experiments, n=73. **(F)** The figure shows the mean ± SEM of BCL-2 expression in splenic hCD45^+^CD19^+^ cells from mice across two independent experiments, n=39. **(G, H)** The figures show the mean ± SEM of MCL-1 **(G)** and BCL-XL **(H)** expression in splenic hCD45^+^CD19^+^ cells from mice from a single experiment performed with cells from a patient with unmutated IGHV, n=19. Statistical analyses were performed using the Kruskal–Wallis test followed by Dunn’s multiple comparisons test, **p<0.01, *p<0.05.

Evaluation of spleen weight (**Figure 3B**) and splenic hCD45^+^CD19^+^CD5^+^ cell numbers across the four treatment groups (**Figure 3C**) revealed no significant differences between venetoclax-treated and control mice. These findings indicate that, in this transfer model, CLL cells exhibit reduced sensitivity to venetoclax induced by the *in vivo* microenvironent that included autologous activated T cells, consistent with the T cell-associated venetoclax resistance observed *in vitro* (13, 14). Opaganib alone also failed to reduce tumor burden. By contrast, the combination of venetoclax and opaganib reduced spleen weight (**Figure 3B**) and splenic CLL-cell counts by ~50% versus controls (**Figure 3C**) (Non-normalized data for **Figure 3C** and the corresponding donor-level statistical analysis are shown in **Supplementary Figures S3A** and **S4**, respectively). When samples were analyzed according to IGHV mutational status, a similar trend was observed, with a seemingly greater reduction in IGHV-unmutated cases. However, neither subgroup analysis reached statistical significance (not shown), likely owing to the limited sample size. Only a few CLL cells were detected in the bone marrow and peripheral blood and did not significantly differ among groups (**Supplementary Figures S5A, B**).

Despite the reduction in splenic tumor load observed upon venetoclax and opaganib combination, splenic hCD45^+^CD19^+^ cells that survived the treatment did not show significant differences in the mean fluorescence intensity (MFI) of the proliferation marker Ki-67 (**Figure 3D**) or the activation marker PD-1 (**Figure 3E**). Likewise, no significant differences in the MFI of BCL-2 were observed among treatment groups when all patient samples were analyzed together (**Figure 3F**). However, stratification by IGHV mutational status revealed a significantly higher BCL-2 expression in the venetoclax-treated groups within the IGHV-mutated subgroup (**Supplementary Figure S6A**). Owing to sample availability, MCL-1 (**Figure 3G**) and BCL-XL expression (**Figure 3H**) could only be assessed in the IGHV-unmutated subgroup, where no significant differences were detected among treatment groups. Similarly, no significant differences were found in the percentage of splenic hCD45^+^CD19^+^ cells expressing BCL-2, MCL-1 and BCL-XL (**Supplementary Figures S6B-D**).

### The impact of venetoclax and opaganib on the T cell compartment in the CLL xenograft model

Our previous *in vitro* studies demonstrated that SPHK inhibitors did not affect T cell survival at 72 h (20) while venetoclax only slightly induced T cell death compared to leukemic cells (13). Regarding our *in vivo* model, no significant differences were observed among groups in hCD45^+^CD3^+^ cell counts when bone marrow and peripheral blood were analyzed (**Supplementary Figures S5C, D**). By contrast, in mice engrafted with IGHV unmutated CLL samples, treatment with the combination of venetoclax and opaganib significantly reduced splenic hCD45^+^CD3^+^ T cell numbers compared with controls, whereas no significant differences were observed in mice engrafted with IGHV mutated CLL samples (**Figure 4A**). Analysis of splenic T cell subsets showed that, in the unmutated cohort, CD4^+^ T cell numbers were significantly reduced in the venetoclax + opaganib group (**Figure 4B**), whereas CD8^+^ T cell numbers were reduced in both opaganib-containing treatment groups (**Figure 4C**). Despite these changes, the CD4^+^/CD8^+^ T cell ratio remained unchanged across treatment groups (**Figure 4D**) (The corresponding non-normalized data for **Figures 4A–C** are provided in **Supplementary Figures S3B–D**).

**Figure 4:**
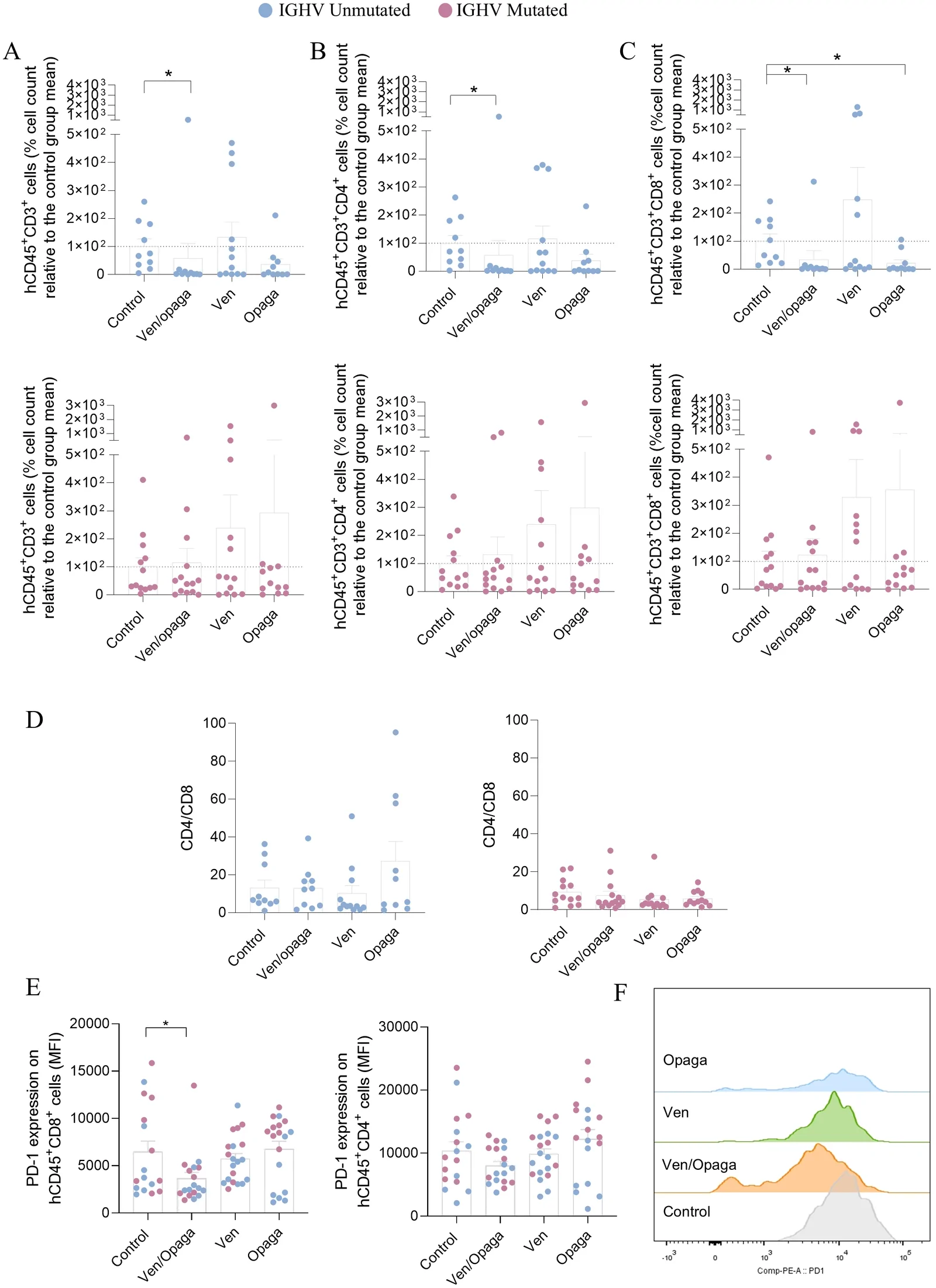
The combination of opaganib and venetoclax modulates the T cell compartment in a CLL xenograft model. Splenic T cells from mice included in the experiments shown in **Figure 3** were subjected to flow cytometric analysis to evaluate the T cell compartment. The figures show the mean ± SEM of the splenic hCD45^+^CD3^+^ **(A)**, hCD45^+^CD4^+^ **(B)**, and hCD45^+^CD8^+^ **(C)** cell count, normalized to the mean of the corresponding control group in each experiment. Data are presented separately for mice engrafted with cells from three patients with unmutated IGHV (upper panels; n=43) and three patients with mutated IGHV (lower panels; n=52). **(D)** The graphs show the mean ± SEM of the CD4^+^/CD8^+^ T-cell ratio from mice engrafted with cells from three patients with unmutated IGHV (n = 43) and from three patients with mutated IGHV (n = 52). **(E)** The figure shows the mean ± SEM of PD-1 expression on splenic hCD45^+^CD3^+^CD8^+^ T cells (left) and hCD45^+^CD3^+^CD4^+^ T cells (right) from mice across four independent experiments (n = 73). **(F)** Representative histograms showing PD-1 expression on splenic CD8^+^ T cells. Statistical analysis was performed using the Kruskal–Wallis test followed by Dunn’s multiple comparisons test, *p<0.05.

As shown in **Figure 4E**, splenic CD8^+^ T cells from the venetoclax + opaganib group had lower PD-1 expression than controls, whereas CD4^+^ T cells did not show a significant change. This suggests that the drug combination not only reduced leukemic burden in the spleen but also altered the T cell compartment, particularly within the CD8^+^ subset. When mutated and unmutated samples were evaluated separately, neither subgroup reached statistical significance, likely because of the limited sample size (**Supplementary Figure S7**). The expression of PD-1 in CD8^+^ cells from a representative sample is shown in **Figure 4F**.

Taken together, our *in vivo* results indicate that opaganib can partially overcome venetoclax resistance in a CLL xenograft model that reproduces microenvironment-mediated drug resistance. The combination of venetoclax and opaganib consistently reduced splenic leukemic burden and was associated with changes in the splenic T cell compartment. These findings provide *in vivo* proof-of-concept supporting SPHK2 inhibition as a strategy to overcome microenvironment-driven venetoclax resistance in CLL.

## Discussion

Our study provides the first *in vivo* evidence that opaganib, an orally bioavailable SPHK2 inhibitor, in combination with venetoclax can reduce microenvironment-driven venetoclax resistance in CLL. Using a xenograft transfer model built by co-transfer of primary CLL cells with autologous activated T cells, we show that venetoclax resistance conferred by the immune microenvironment is faithfully reproduced *in vivo*, that opaganib alone is insufficient to reduce leukemic burden but in combination with venetoclax reduces splenic tumor load by approximately 50% while simultaneously reshaping the tumor T cell compartment.

Our data also further refine our *ex vivo* model of T cell-mediated venetoclax resistance (14) and the current understanding of T cell-CLL interactions by demonstrating that CD4^+^ T cells, but not CD8^+^ T cells, are responsible for inducing venetoclax resistance. This observation is consistent with previous studies identifying CD4^+^ T cells as the principal immune population sustaining CLL growth and survival *in vivo* (34, 35). Moreover, in the xenograft model used, autologous CD4^+^ T cells are indispensable for leukemic-cell proliferation, whereas depletion of CD8^+^ T cells have little impact, highlighting a non-redundant role for helper T cells in supporting disease progression (28). Likewise, genetic and depletion studies in the Eμ-TCL1 model of CLL revealed distinct roles for murine T cell subsets, with CD4^+^ T cells supporting leukemic expansion, whereas CD8^+^ T cells restrain disease progression (36).

Our data also demonstrate that CD4^+^ T cell-derived EVs are sufficient to generate venetoclax resistant CLL cells. These findings support a model in which activated CD4^+^ T cells orchestrate multiple complementary mechanisms that sustain CLL persistence and limit the efficacy of BCL-2 inhibition. The role of EVs in CLL has received increasing attention over the past decade, largely focusing on vesicles released by leukemic cells or stromal components of the tumor microenvironment. These studies have shown that CLL-derived EVs educate stromal cells, endothelial cells and monocytes to establish a permissive niche that favors leukemic-cell survival, immune evasion and disease progression (19, 37). In addition, CLL-derived EVs have been shown to reprogram T cells toward dysfunctional phenotypes characterized by impaired immune synapse formation, altered cytokine production and reduced cytotoxic function, by promoting CD8^+^ T cell exhaustion and impairing T cell activation and metabolic fitness through multiple immune checkpoint pathways, further reinforcing a leukemia-supportive microenvironment (18, 19). Conversely, recent reports have demonstrated that engineered CAR T cell-derived EVs can exert potent antileukemic activity (38, 39), supporting the concept that vesicle-mediated communication can also be therapeutically exploited. However, whether physiological, non-engineered T cell-derived EVs directly modulate leukemic cell drug sensitivity has remained largely unexplored.

Our findings fill this gap by demonstrating that activated helper T cells can transfer resistance-inducing signals through EVs, thereby extending the concept of bidirectional communication within the CLL immune microenvironment. Future studies should determine the molecular cargo of EVs derived from T cells of CLL patients responsible for this effect and establish its contribution to therapeutic resistance in patients. Given that CD40L is expressed on CD4^+^ T cell-derived EVs (**Supplementary Figure S1B**), it is intriguing to postulate that EVs can recapitulate, at least in part, the canonical helper T cell-CLL cell interaction. By delivering membrane-bound CD40L to leukemic cells, these vesicles could sustain CD40-dependent prosurvival signaling independently of direct cell-cell contact, thereby contributing to venetoclax resistance. Future studies will be required to determine the functional relevance of this pathway. Moreover, an important question that remains unresolved is how EV-mediated interactions operate in CLL patients. While, as mentioned above, CLL-derived EVs have been shown to impair T cell function and promote immune dysfunction (18, 19), our data indicate that T cell-derived EVs, in turn, promote leukemic-cell activation and drug resistance. Understanding how these opposing vesicle-mediated signals are integrated within the lymphoid microenvironment, and whether one pathway predominates during disease progression or treatment, will be important for defining the overall contribution of EV-mediated communication to CLL biology and therapeutic response.

Regarding our *in vitro* model of T cell-driven venetoclax resistance, it relied on short-term *in vitro* co-culture systems (20), which cannot recapitulate the spatial organization, cellular dynamics and tissue-specific interactions of the lymphoid microenvironment. By co-engrafting primary CLL cells with autologous activated T cells, the CLL xenograft model captures key aspects of these patient-specific immune interactions, providing a more physiologically relevant platform to evaluate therapeutic strategies. Using this model, we demonstrate that venetoclax resistance driven by the immune microenvironment can be reproduced *in vivo*. Opaganib alone exerted minimal antileukemic activity in the xenograft model, whereas in combination with venetoclax significantly reduced splenic leukemic burden, indicating that SPHK2 inhibition potentiates the activity of BCL-2 blockade. Although the antileukemic effect of the combination appeared more pronounced in mice engrafted with IGHV-unmutated CLL samples, subgroup analyses did not reach statistical significance because of the limited sample size. This observation should therefore be considered exploratory and warrants future evaluation in larger cohorts to determine whether the efficacy of opaganib differs according to IGHV mutational status. Given the short duration of the xenograft experiments, the emergence of genetically acquired resistance mechanisms, such as BCL2 mutations, is highly unlikely, suggesting that the reduced sensitivity to venetoclax primarily reflects adaptive microenvironment-dependent mechanisms. Collectively, our study establishes this widely used CLL xenograft model as a valuable preclinical platform for studying microenvironment-driven venetoclax resistance *in vivo* and provides the first *in vivo* evidence that opaganib in combination with venetoclax can partially overcome this resistance, extending our previous *ex vivo* observations into a clinically relevant preclinical model.

An intriguing finding of the present study was that, despite the marked reduction in splenic leukemic burden upon venetoclax + opaganib treatment, residual CLL cells recovered from treated mice did not significantly differ in the expression of the proliferation marker Ki-67 or the activation marker PD-1 compared to control or other treatment groups. Moreover, based on our previous *ex vivo* studies, we hypothesized that the restoration of venetoclax sensitivity by opaganib would be accompanied by a reduction in the expression of anti-apoptotic BCL-2 family proteins that are not directly targeted by venetoclax. However, the therapeutic benefit of the combination was not accompanied by a particular expression of MCL-1 or BCL-XL in the surviving leukemic cells, indicating that the therapeutic activity of opaganib *in vivo* cannot be fully explained by changes in the expression of these molecules and suggesting that additional mechanisms contribute to the restoration of venetoclax sensitivity. Beyond MCL-1 and BCL-XL, A1/BFL-1 represents another potential mechanism of BH3-mimetic resistance. Thus, the proapoptotic protein BIM may interact with anti-apoptotic BCL-2 members in a sequential manner in the order of BCL-2, BCL-XL, MCL-1, and, lastly, A1/BFL-1, revealing a functional hierarchy of antiapoptotic Bcl-2 members in determining resistance to venetoclax (9). Moreover, alterations affecting the anti-apoptotic protein A1/BFL-1 or pro-apoptotic molecules, including subclonal losses of BAX or NOXA, have also been described in single-cell multiomic profiling of CLL cells relapsing after venetoclax (10). Because our analysis did not assess A1/BFL-1, BAX, or NOXA, further studies incorporating the broader BCL-2 family network and functional BH3 profiling will be required to determine whether these pathways contribute to the restoration of venetoclax sensitivity by opaganib.

In addition to its canonical role in sphingolipid metabolism (40), SPHK2 has also been proposed to exert a pro-apoptotic activity, partially independent on its kinase activity, through a BH3-like motif that mediates direct interaction with pro-survival BCL-2 family members, including BCL-XL and BCL-2 (41, 42). Consistent with this observation, mutation of the conserved Leu-219 within this BH3-like motif abolishes BCL-XL interaction and SPHK2-induced apoptosis (41). Although the relevance of this mechanism in CLL cells remains unknown, it may represent an additional mechanism through which pharmacological inhibition of SPHK2 by opaganib favors protein-protein interactions and apoptotic priming without necessarily altering BCL-XL or MCL-1 expression.

Moreover, while initially developed as a selective SPHK2 inhibitor that reduces S1P production (43), opaganib is now recognized as a broader modulator of sphingolipid metabolism through its additional effects on dihydroceramide desaturase (DES1) and glucosylceramide synthase (GCS) (44). Consequently, it reduces intracellular S1P levels while increasing ceramide and dihydroceramide accumulation, leading to suppression of key pro-survival signaling pathways such as ERK, AKT, and NF-κB and promoting autophagy and/or apoptosis in different solid and hematological neoplasm (44). Whether any of these additional biological activities contribute to the restoration of venetoclax sensitivity observed in our model warrants further investigation.

Finally, preclinical studies have shown that SPHK2 inhibition can enhance antitumor immunity by promoting immunogenic cell death and response to immune checkpoint blockade (26) and by acting on non-malignant cells from the tumor microenvironment. Thus, the inhibition of SPHK2 favors immunogenic differentiation of myeloid derived suppressor cell (MDSC) mitigating their suppressive functions (45) and enhances anti-tumor immunity in a mouse model of multiple myeloma by promoting CD8 T cell activation (46). In our xenograft model, mice treated with venetoclax + opaganib showed selective remodeling of the splenic T cell compartment. The combination reduced the absolute number of CD4^+^ T cells and CD8^+^ T cells in unmutated CLL samples while simultaneously decreasing PD-1 expression within the remaining CD8^+^ population. The present data do not allow us to distinguish whether the reduction in PD-1 expression among residual CD8^+^ T cells reflects preferential contraction of a dysfunctional PD-1^high^ subset following SPHK2 inhibition by opaganib and/or a partial reversal of the exhausted phenotype. The latter possibility is supported by previous evidence linking S1P signaling to impaired CD8^+^ T-cell metabolism, effector function, and increased susceptibility to apoptosis (47), together with increased numbers of granzyme B- and IFN-γ-producing CD8^+^ T cells reported following opaganib treatment in a phase II clinical trial in patients with metastatic castration-resistant prostate cancer (NCT04207255) (45). Although these findings raise the possibility that opaganib may restore aspects of CD8^+^ T-cell function, further studies are warranted to determine whether the reduction in PD-1 expression observed in our model reflects functional reprogramming of CD8^+^ T cells or changes in the composition of the residual T-cell population. This distinction is particularly relevant given the central role of the CLL lymph node microenvironment in sustaining disease, where multiple T cell subsets, including PD-1^+^ CD8^+^ T cells, contribute to the complex cellular interactions that support CLL survival and persistence (48). Defining how opaganib affects these T cell populations and their interactions with CLL cells will therefore be important to determine whether its activity disrupts immune mechanisms contributing to venetoclax resistance and thereby restores drug sensitivity.

In conclusion, these findings support the combination of venetoclax and opaganib as a rational strategy to overcome microenvironment-driven resistance in CLL and provide a preclinical rationale for its evaluation in patients whose disease is shaped by an active immune microenvironment.

## Data Availability

The original contributions presented in the study are included in the article/**Supplementary Material**. Further inquiries can be directed to the corresponding author.
