# Supplementary material for: T cell–driven venetoclax resistance in chronic lymphocytic leukemia: in vivo preclinical activity of opaganib
Source: Front Immunol. 2026 Oct 15;17:1968413. doi: 10.3389/fimmu.2026.1968413 (PMC13717244; doi:10.3389/fimmu.2026.1968413)
Supplement: Supplementary file 1 [file DataSheet1.pdf]

SUPPLEMENTARY FIGURES

Supplementary Figure S 1

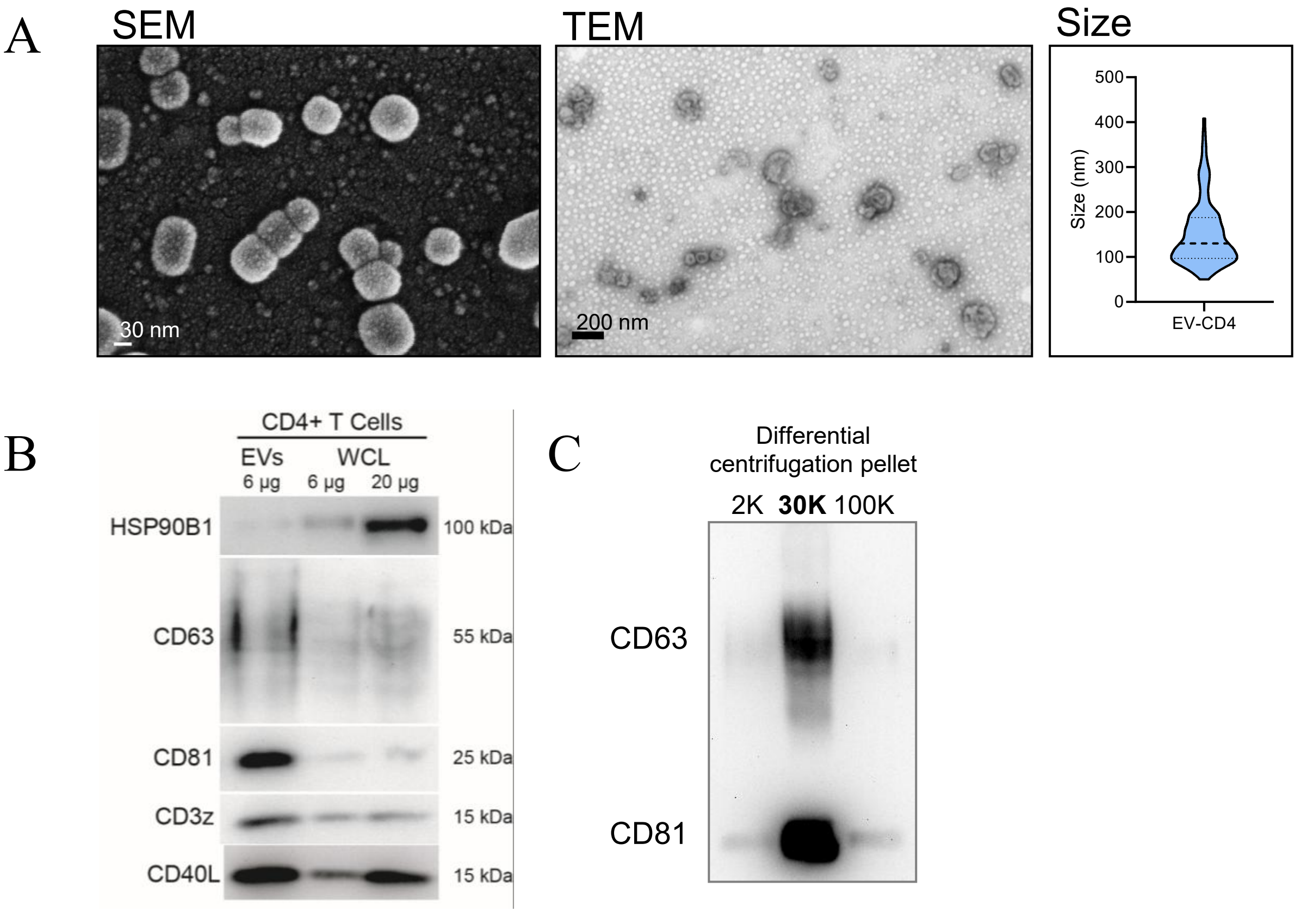

Supplementary FIGURE S1. EVs produced by activated CD4+T cells were characterized by electron microscopy and immunoblot.

(A) Micrographs of scanning (SEM) and transmission (TEM) electron microscopy depicting characteristic EV-structures; EV-size was determined on TEM micrographs using ImageJ software measuring 265 EV-compatible images.

(B) Immunoblot characterization of EVs compared to the whole cell lysate (WCL); tetraspanins CD63 and CD81 were enriched in EVs, while the ER-resident protein HSP90B1 was excluded; T cell proteins CD3z and CD40L were detected on EVs.

(C) Validation immunoblot showing that the EV-markers CD63 and CD81 were completely pelleted by centrifugation at 30000 g (30K), leaving scarce remnant material in the subsequent 100000 g centrifugation (100K).

Supplementary Figure S 2

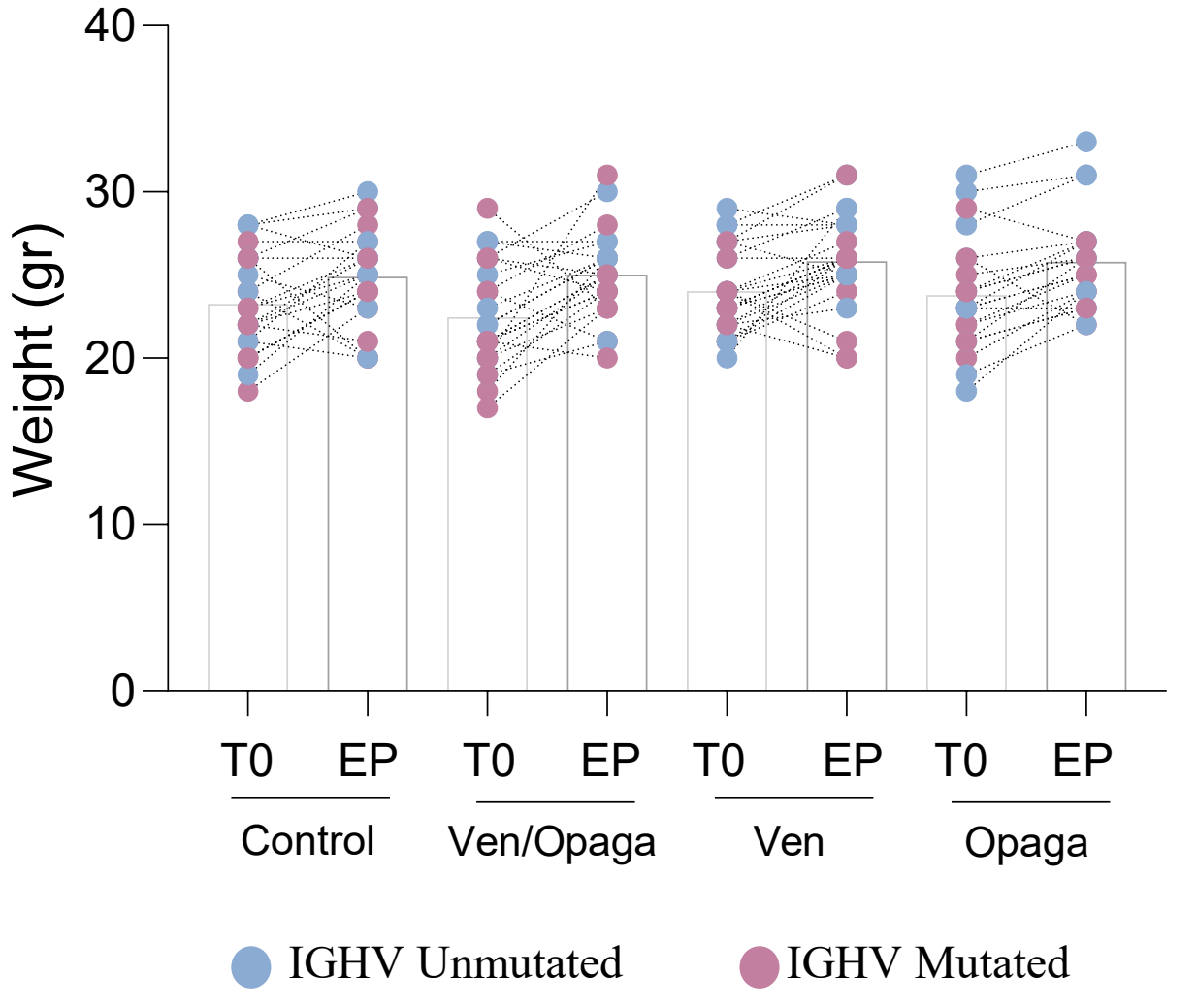

Supplementary Figure S 2: Mice from all four groups were weighed 24 h before treatment initiation (T0) and at the endpoint (EP). Statistical analyses were performed using the Kruskal-Wallis test followed by Dunn's multiple comparisons test. N=95

Supplementary Figure S 3

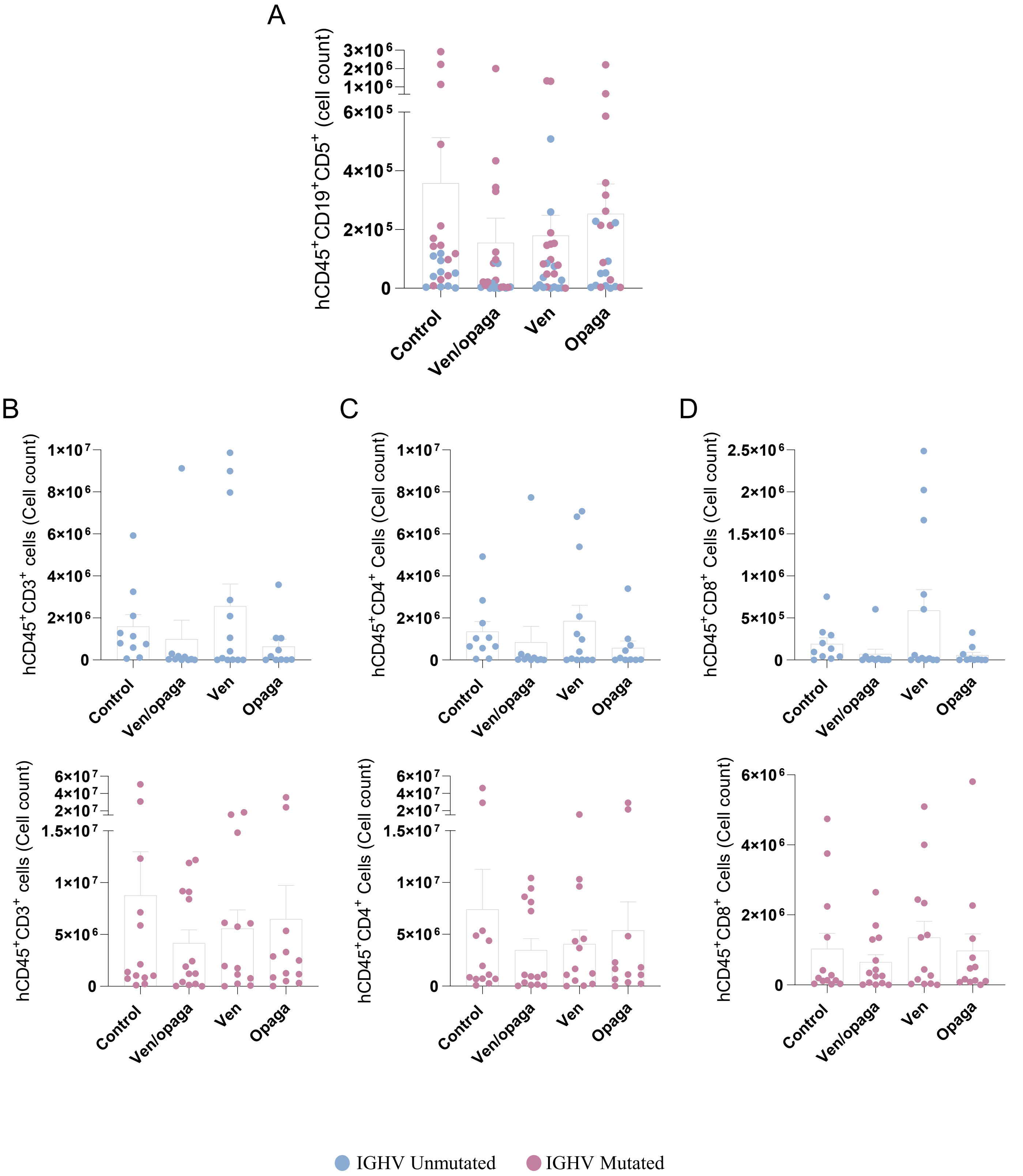

Supplementary FIGURE S3. **Absolute cell numbers of splenic B cells and T cells from control and treated mice.**

(A) The graph shows the mean ± SEM of absolute number of $hCD45^+CD19^+CD5^+$cells, n=95.

(B) The figures show the mean ± SEM of the absolute number of splenic $hCD45^+CD3^+$ cells.

(C) The figures show the mean ± SEM of the absolute number of splenic $hCD45^+CD4^+$ cells.

(D) The figures show the mean ± SEM of the absolute number of splenic $hCD45^+CD8^+$cells.

Data from sections B-D are presented separately for mice engrafted with cells from three patients with unmutated IGHV (n=43) and three patients with mutated IGHV (n=52). Statistical analyses were performed using the Kruskal-Wallis test followed by Dunn's multiple comparisons test.

Supplementary Figure S 4

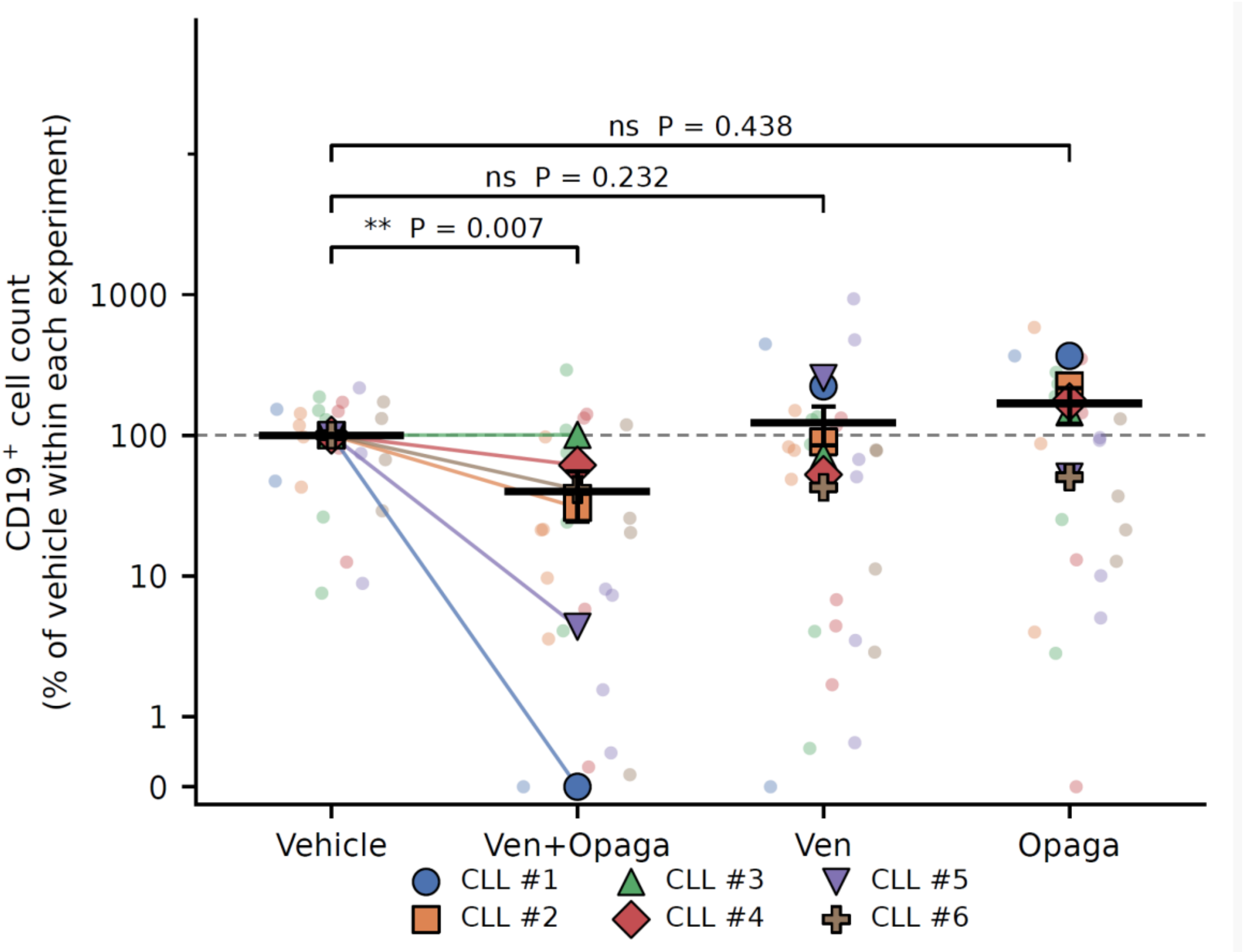

Supplementary Figure S4. Donor-level statistical analysis of splenic CLL-cell counts. The figure shows small translucent symbols that correspond to individual mice, colour-coded by donor of origin; large outlined symbols are the mean of each donor, with lines connecting the vehicle and combination arms of the same donor; black bars are the mean ± SEM of the six donor means. Counts are expressed as a percentage of the vehicle arm of the same experiment (dashed line, 100%). The y axis is symmetrical-logarithmic to accommodate three animals with no CD19+ cells detected, plotted at 0. P values were calculated using a linear mixed-effects model with donor included as a random intercept and adjusted for multiple comparisons using the Holm method. ns, not significant; **P < 0.01.

Supplementary Figure S 5

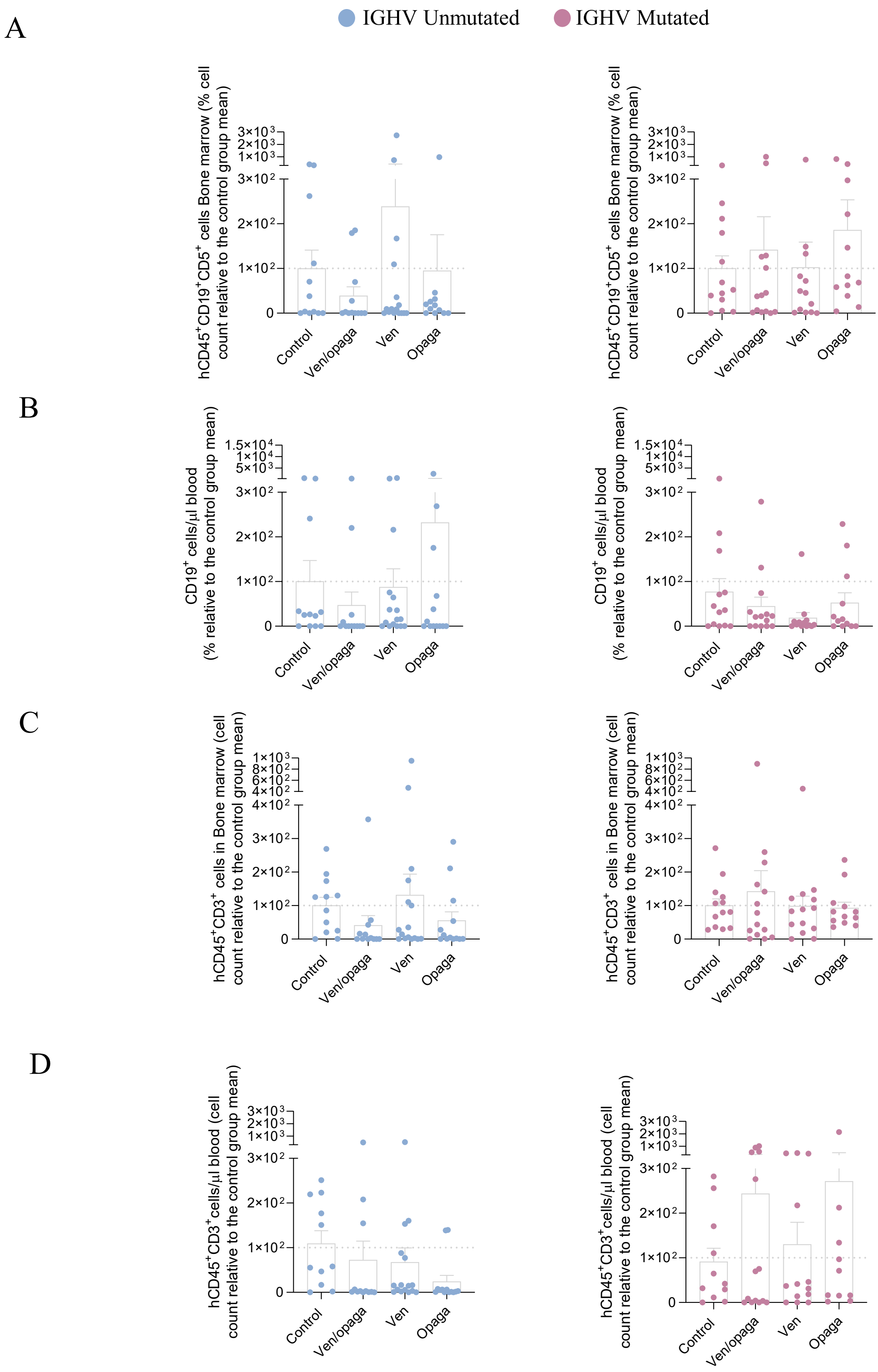

Supplementary FIGURE S5. Bone marrow and peripheral blood from control and treated mice.

(A) The graphs show the mean ± SEM of $hCD45^+CD19^+CD5^+$ cells count in bone marrow relative to the mean of the control group.

(B) The graphs show the mean ± SEM of $hCD45^+CD19^+CD5^+$ cells count per microliter of blood in peripheral blood relative to the mean of the control group.

(C) The graphs show the mean ± SEM of $hCD45^+CD3^+$ cells count in bone marrow relative to the mean of the control group.

(D) The graphs show the mean ± SEM of $hCD45^+CD3^+$ cells count per microliter of blood in peripheral blood relative to the mean of the control group.

Data are presented separately for mice engrafted with cells from three patients with unmutated IGHV (n=43) and three patients with mutated IGHV (n=52). Statistical analyses were performed using the Kruskal–Wallis test followed by Dunn's multiple comparisons test.

Supplementary Figure S 6

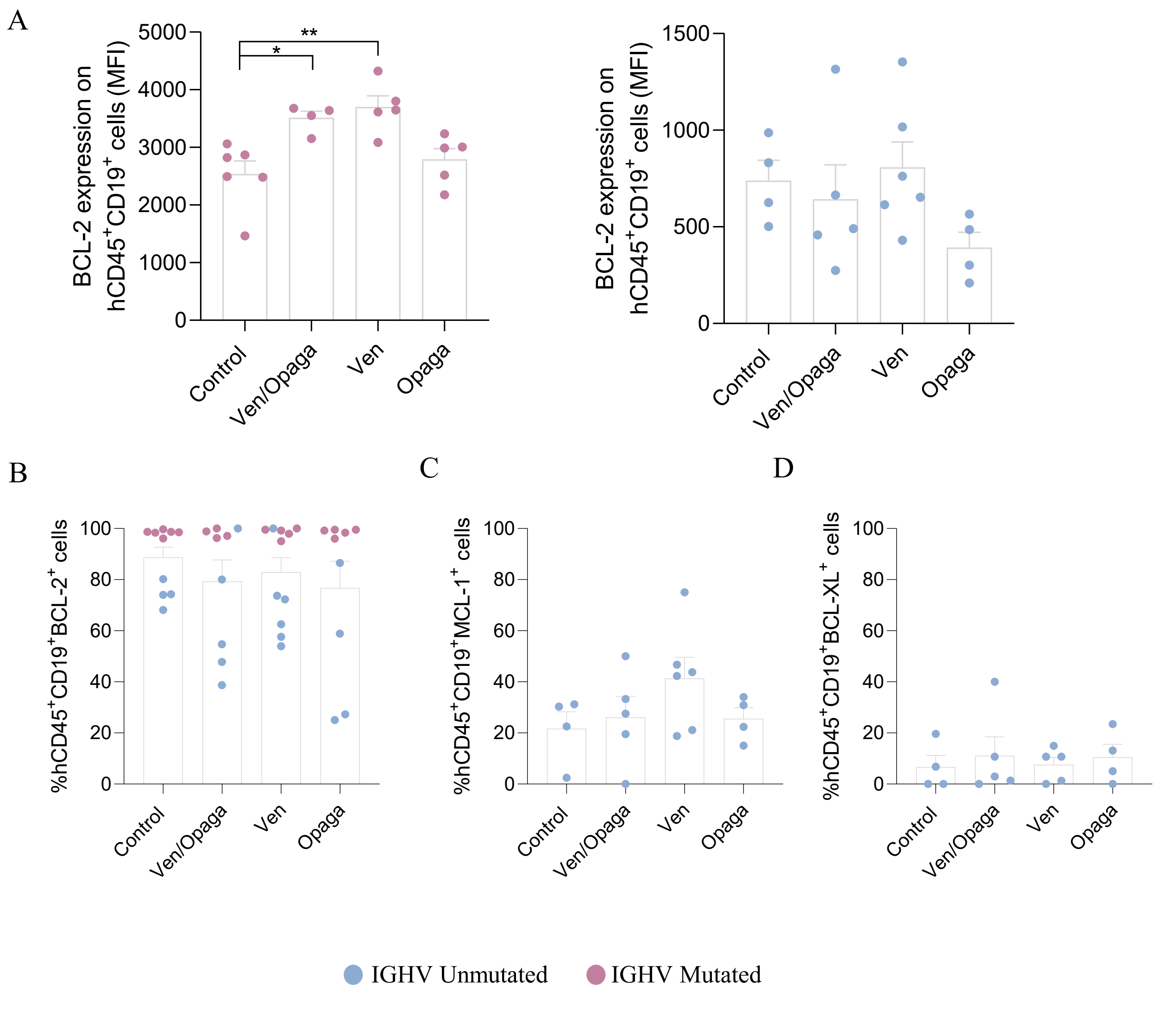

Supplementary FIGURE S6. The expression of BCL-2 family members in splenic $hCD45^+CD19^+$ cells.

(A) The figure shows the mean ± SEM of BCL-2 expression in splenic $hCD45^+CD19^+$ cells from mice across two independent experiments, one using cells from a patient with mutated IGHV (right, n=19) and the other with cells from a patient with unmutated IGHV (left, n=20).

(B) The graphs show the percentage of splenic $hCD45^+CD19^+BCL\text{-}2^+$ cells. BCL-2 was evaluated in two independent experiments, one using cells from a patient with mutated IGHV and the other with cells from a patient with unmutated IGHV, n=39.

(C) The graphs show the percentage of splenic $hCD45^+CD19^+MCL\text{-}1^+$ cells. MCL-1 was assessed in a single experiment performed with cells from a patient with unmutated IGHV, n=19.

(D) The graphs show the percentage of splenic $hCD45^+CD19^+BCL\text{-}XL^+$ cells. BCL-XL was assessed in a single experiment performed with cells from a patient with unmutated IGHV, n=19.

Statistical analyses were performed using the Kruskal–Wallis test followed by Dunn's multiple comparisons test, $^{**}p<0.01$ $^{*}p<0.05$.

Supplementary Figure S 7

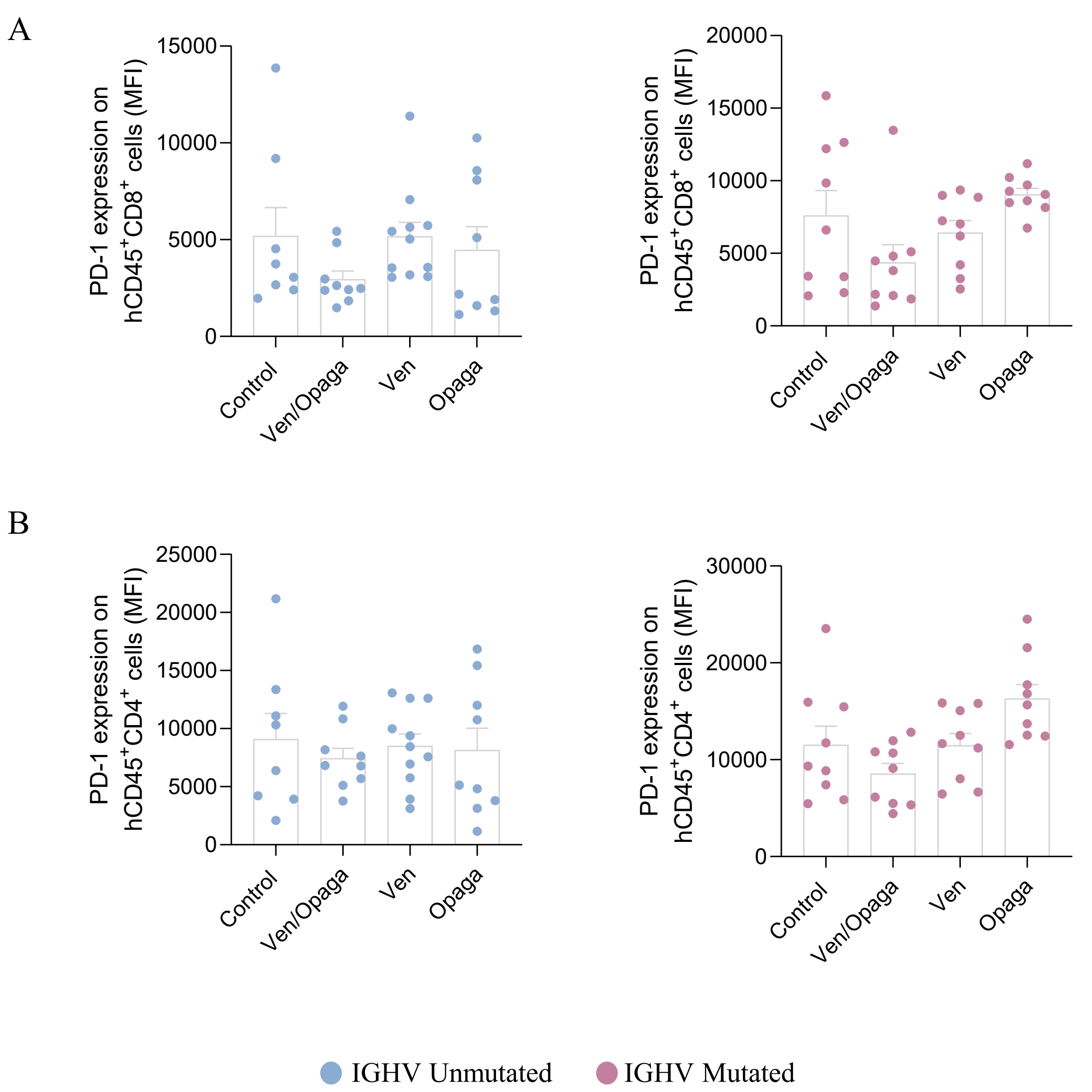

Supplementary FIGURE S7. PD-1 expression on splenic T cell subsets from control and treated mice.

(A) The figure shows the mean ± SEM of PD-1 expression on splenic $hCD45^+CD3^+CD8^+$ T cells

(B) The figure shows the mean ± SEM of PD-1 expression on splenic and $hCD45^+CD3^+CD4^+$ T cells.

Mice from four independent experiments were included, employing cells from two patients with unmutated IGHV (n=37) and two patients with mutated IGHV (n=36). Statistical analysis was performed using the Kruskal–Wallis test followed by Dunn's multiple comparisons test.
