# Supplementary material for: T cell–driven venetoclax resistance in chronic lymphocytic leukemia: in vivo preclinical activity of opaganib
Source: Front Immunol. 2026 Oct 15;17:1968413. doi: 10.3389/fimmu.2026.1968413 (PMC13717244; doi:10.3389/fimmu.2026.1968413)
Supplement: Supplementary file 2 [file DataSheet2.pdf]

Supplementary Table 1: distribution of mice per CLL donor and per treatment group*

CLL donor	Vehicle	Ven+opaga	Ven	opaga	Total
CLL #1	2	1	2	1	**6**
CLL #2	4	5	4	3	**16**
CLL #3	5	5	5	5	**20**
CLL #4	5	5	5	5	**20**
CLL #5	4	4	6	4	**18**
CLL #6	4	4	4	4	**16**
**Total**	**24**	**24**	**26**	**22**	**96**

* One mouse was lost during the oral gavage procedure in Experiment with CLL # 5 within vehicle arm. Overall, 95 animals were ultimately included in the analysis.
